# Late Thrombectomy in Clinical Practice: Retrospective Application of DAWN/DEFUSE3 Criteria within the German Stroke Registry

**DOI:** 10.1007/s00062-021-01033-1

**Published:** 2021-06-07

**Authors:** Moriz Herzberg, Korbinian Scherling, Robert Stahl, Steffen Tiedt, Frank A. Wollenweber, Clemens Küpper, Katharina Feil, Robert Forbrig, Maximilian Patzig, Lars Kellert, Wolfgang G. Kunz, Paul Reidler, Hanna Zimmermann, Thomas Liebig, Marianne Dieterich, Franziska Dorn, GSR investigators, T. Boeckh-Behrens, S. Wunderlich, A. Reich, M. Wiesmann, U. Ernemann, T. Hauser, E. Siebert, C. Nolte, S. Zweynert, G. Bohner, A. Ludolph, K.-H. Henn, W. Pfeilschifter, M. Wagner, J. Röther, B. Eckert, J. Berrouschot, C. Gerloff, J. Fiehler, G. Thomalla, A. Alegiani, E. Hattingen, G. Petzold, S. Thonke, C. Bangard, C. Kraemer, M. Dichgans, M. Psychogios, J. Liman, M. Petersen, F. Stögbauer, P Kraft, M. Pham, M. Braun, A. Kastrup, K. Gröschel, T. Uphaus, V. Limmroth

**Affiliations:** 1 Institute of Neuroradiology, Ludwig Maximilians University (LMU) Munich, Campus Grosshadern, Marchioninistraße 15, 81377 Munich, Germany; 2 Department of Radiology, University Hospital, Würzburg, Germany; 3 Institute for Stroke and Dementia Research, University Hospital, LMU Munich, Munich, Germany; 4 Department of Neurology, Hospital Wiesbaden, Wiesbaden, Germany; 5 Department of Neurology, University Hospital, LMU Munich, Munich, Germany; 6 Department of Neurology, University Hospital, Tübingen, Germany; 7 Department of Radiology, University Hospital, LMU Munich, Munich, Germany; 8 Department of Neuroradiology, University Hospital, Bonn, Germany

**Keywords:** Stroke, Endovascular therapy, Outcome, Late thrombectomy

## Abstract

**Background and Purpose:**

To provide real-world data on outcome and procedural factors of late thrombectomy patients.

**Methods:**

We retrospectively analyzed patients from the multicenter German Stroke Registry. The primary endpoint was clinical outcome on the modified Rankin scale (mRS) at 3 months. Trial-eligible patients and the subgroups were compared to the ineligible group. Secondary analyses included multivariate logistic regression to identify predictors of good outcome (mRS **≤** 2).

**Results:**

Of 1917 patients who underwent thrombectomy, 208 (11%) were treated within a time window ≥ 6–24 h and met the baseline trial criteria. Of these, 27 patients (13%) were eligible for DAWN and 39 (19%) for DEFUSE3 and 156 patients were not eligible for DAWN or DEFUSE3 (75%), mainly because there was no perfusion imaging (62%; *n* = 129). Good outcome was not significantly higher in trial-ineligible (27%) than in trial-eligible (20%) patients (*p* = 0.343). Patients with large trial-ineligible CT perfusion imaging (CTP) lesions had significantly more hemorrhagic complications (33%) as well as unfavorable outcomes.

**Conclusion:**

In clinical practice, the high number of patients with a good clinical outcome after endovascular therapy ≥ 6–24 h as in DAWN/DEFUSE3 could not be achieved. Similar outcomes are seen in patients selected for EVT ≥ 6 h based on factors other than CTP. Patients triaged without CTP showed trends for shorter arrival to reperfusion times and higher rates of independence.

**Supplementary Information:**

The online version of this article (10.1007/s00062-021-01033-1) contains supplementary material, which is available to authorized users.

## Introduction

The results of the DAWN and DEFUSE3 trials have extended the indications for endovascular therapy (EVT) in selected stroke patients in late time intervals [1–3]. The DAWN trial enrolled patients last seen well between 6 and 24 h before admission and with a discrepancy between the clinical deficit (National Institutes of Health Stroke Scale [NIHSS]) ≥ 10; ≥ 20) and the size of the presumed infarct core on advanced perfusion imaging (core volume < 21 ml; ≥ 31–< 51 ml). The DEFUSE3 trial had less strict criteria than DAWN (approximately 40% of the patients in DEFUSE3 did not meet the DAWN selection criteria) [3]. All patients had expected core infarcts of less than 70 ml as determined by diffusion-weighted magnetic resonance imaging (DWI) or computed tomography perfusion imaging (CTP) (median ischemic core volume 9.4 ml vs. 7.6 ml in DAWN trial). Clinical benefit was independently demonstrated for the subgroups of patients who met either DAWN or DEFUSE3 eligibility criteria [4, 5]. Subsequently, the current guidelines recommended EVT in the late time window if patients meet either DAWN or DEFUSE3 trial criteria [1, 6]. Whether the trial results are transferable to clinical routine remains an open issue [7–9].

The German Stroke Registry (GSR) is a prospective, academic, industry-independent registry established to evaluate real-world outcomes of EVT on a multicentric nationwide scale [10, 11].

We retrospectively analyzed trial-eligible patients and compared clinical outcome and procedural factors to trial-ineligible patients to create a real-world context for the application of the trial criteria.

## Methods

### Study Population

All included patients were part of the GSR (clinical trial registration URL: http://www.clinicaltrials.gov. Unique identifier: NCT03356392) between June 2015 and April 2018. The 25 participating centers reflect both primary and comprehensive stroke centers (12 university hospitals and 13 municipal hospitals). A detailed description of the GSR study design, obtained parameters, and main outcome data was recently published [9, 10].

We retrospectively included patients from the GSR cohort with a complete dataset and Large Vessel Occlusion (LVO). Patients were dichotomized according to the inclusion criteria in the RCTs:

- DAWN/DEFUSE3-like (DD)
  A. ≥ 80 years, NIHSS ≥ 10, infarction core < 21 ml
  B. < 80 years, NIHSS ≥ 10, infarction core < 31 ml
  C. < 80 years, NIHSS ≥ 20, infarction core ≥ 31 ml +< 51 ml
  D. ≥ 18–90 years, NIHSS ≥ 6, infarction core < 70 ml, penumbra ≥ 15 ml, mismatch ratio > 1.8
- Non-DAWN, non-DEFUSE3-like (NDND)
- All patients NIHSS ≥ 6 who underwent EVT 6–24 h

### Image Acquisition and Analysis

Local imaging protocols varied but included at least a non-contrast-enhanced CT (NCCT) scan and a CT angiogram (CTA) for baseline imaging. The CT perfusion imaging (CTP) and magnetic resonance imaging (MRI) with and without perfusion could be used additionally. Evaluation of Alberta Stroke Program Early CT Score (ASPECTS) and modified treatment in cerebral ischemia (mTICI) score was performed by the local neuroradiologists. Infarct volumes were centrally measured for all patients by K. Scherling and M. Herzberg on CTP or MRI by using the approach of RAPID Software (iSchemaView) [12] (detailed description in the online-only data supplement).

### Treatment

All patients underwent EVT and, in some cases, additional recombinant tissue plasminogen activator (rtPA) IV at the discretion of the responsible neurologist. The EVT was performed at the discretion of the neurointerventionalists with direct aspiration and/or a retrievable thrombectomy device. For patients with a tandem stenosis angioplasty with or without stenting could be performed at the neurointerventionalists’ discretion.

### Outcome and Safety

The primary outcome was assessed by trained neurologists, either by face-to-face or telephone interviews using the modified Rankin scale (mRS) at 90 days. Good outcome was defined as mRS 0–2 or back to baseline (*n* = 1 baseline mRS > 2). Secondary outcomes included technical recanalization success (mTICI ≥ 2b), shift in mRS at 90 days, intracerebral hemorrhage (ICH) within 24 h of thrombectomy and death by 90 days.

### Statistics

Differences in quantitative data were evaluated using one-way analysis of variance (ANOVA) and Kruskal-Wallis tests, according to the normal distribution assessed by the Shapiro–Wilk test. Pairwise post hoc tests were conducted with either t‑tests or Mann-Whitney U tests and χ^2^-tests were used to compare qualitative values. For post hoc comparisons pairwise 2 × 2 tables were created. Bonferroni correction was applied to all post hoc tests.

The association between clinical variables and outcome at day 90 was tested using univariate regression analysis. Significant determinants were included in multivariate regression analysis. Analyses were performed using R version 3.6.1 (R Foundation for Statistical Computing, Vienna, Austria) and SPSS Version 25.0 (IBM SPSS Statistics for Windows, IBM Corp. Armonk, NY, USA). A level of significance of alpha = 0.05 was used. Details on statistical analysis are provided in the online-only data supplement.

### Ethics Statement

The GSR-study was approved by the ethics committee of the Ludwig Maximilians University Munich, Germany (689-15) as the leading ethics committee and all local committees following the declaration of Helsinki.

### Data Availability

Anonymized data will be shared by request from any qualified investigator.

## Results

### Patient Selection

**Fig. 1:**
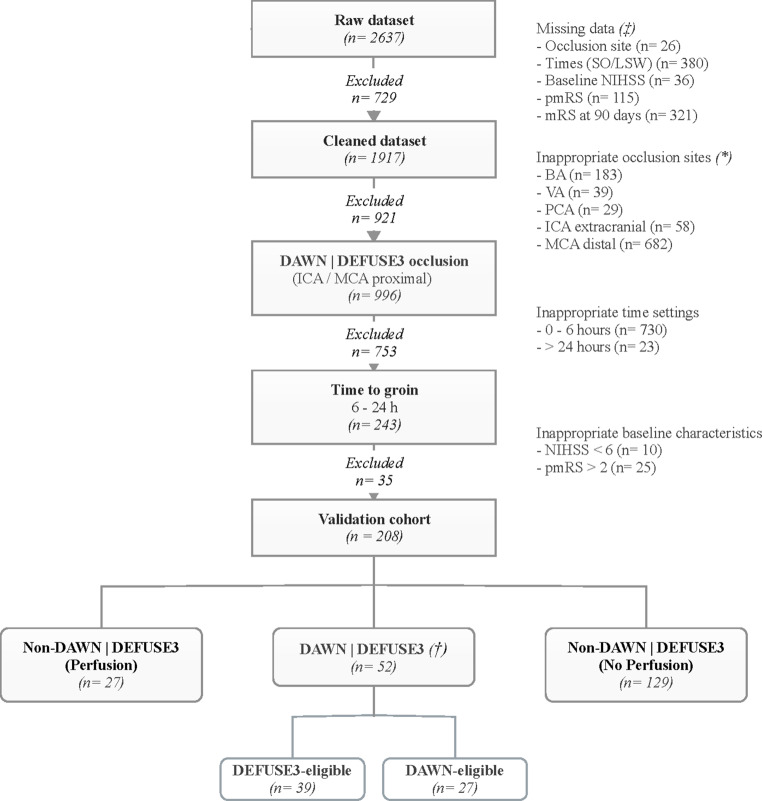
Flowchart shows patient selection and exclusion criteria. *BA* basilar artery, *ICA* internal carotid artery, *LSW* last seen well, *MCA* middle cerebral artery, *mRS* modified Rankin scale, *NIHSS* National Institutes of Health Stroke Scale, *PCA* posterior cerebral artery, *pmRS* premorbid modified Rankin scale, *SO* symptom onset. ‡ multiple missing data possible, * multiple occlusion sites possible, † note the overlap of 14 patients

The main outcome of all patients included in the GSR was recently published. Of the 2637 patients, we excluded all patients according to our selection criteria as illustrated in Fig. 1. Of the 2637 patients, we excluded 729 patients because of incomplete datasets, thus leaving 1917 patients. Another 921 (48%) patients were excluded with artery occlusion types which were not eligible for DAWN and/or DEFUSE3 study criteria (posterior circulation or peripheral MCA-occlusions). Additional 753 (40%) patients presented within a time window below 6 h (*n* = 730) or after more than 24 h (*n* = 23). Furthermore, 35 patients had either an NIHSS < 6 (*n* = 10) or a pmRS > 2 (*n* = 25). Finally, 208 (11%) patients constituted the study population. Most (*n* = 181, 87%) patients presented within the 6–16 h time window, 27 between 16–24 h (13%). The imaging and clinical criteria of DAWN and DEFUSE3 were applied to these 208 patients. Overall, 52 patients (25%) were identified who met the trial criteria (DAWN/DEFUSE3-eligible = DD) and 156 (75%) who did not (non-DAWN/non-DEFUSE3-eligible = NDND). Of the patients 27 (13%) met the DAWN criteria and 39 patients (19%) the DEFUSE‑3 criteria, resulting in an overlap of 14 patients (7%). Within group NDND, 27 patients were labelled as subgroup Non-DAWN/Non-DEFUSE3 with perfusion (NDNDwP) with an ischemic core > 70 ml (*n* = 10, 6.3%), absence of penumbra > 15 ml (*n* = 14, 6.7%) or mismatch ratio lower than 1.8 (*n* = 12, 5.8%). Note the possibility of multiple exclusion criteria. Subgroup Non-DAWN/Non-DEFUSE3 no perfusion (NDNDnP) contained 129 patients with missing or inadequate (*n* = 14) perfusion imaging (62%). Of all patients with perfusion imaging, 52/79 (66%) met DAWN/DEFUSE3 criteria.

### Baseline Characteristics

Detailed baseline characteristics and treatment details are shown in Table 1.

**Table 1:** Baseline characteristics and treatment details^b^

Characteristics	DAWN DEFUSE *eligible* (*n* = 52)	NON-DAWN NON-DEFUSE (with *perfusion*) (*n* = 27)	NON-DAWN NON-DEFUSE (no *perfusion*) (*n* = 129)	Overall *p*-value
*Age—years (SD)*	72.85 (11.56)	75.56 (13.08)	72.29 (12.66)	0.465*
*Age* *≥* *80 years, n (%)*	17 (32.7)	11 (40.7)	42 (32.6)	0.751***
*Sex—female, n (%)*	27 (51.9)	13 (48.1)	74 (57.4)	0.596***
*pmRS median (IQR)*	0 (0.0)	0 (0.1)	0 (0.1)	0.296**
0 (*n*, %)	40 (76.9)	17 (63.0)	96 (74.4)	0.395***
1 (*n*, %)	8 (15.4)	4 (14.8)	18 (14.0)	0.968***
2 (*n*, %)	4 (7.7)	6 (22.2)	15 (11.6)	0.166***
*Baseline NIHSS*				
Median (IQR)	16 (14–19)	16 (13.5–18)	16 (13–20)	0.820**
*Drip- and ship, n (%)*	11 (21.2)	5 (18.5)	39 (30.2)	0.186***
*LSW to admission (minutes)*				
Median (IQR)	616 (530–918)	804 (546–993)	638 (481–851)	0.228**
*Admission to revascularization (minutes)*				
Median (IQR)	141 (98–205)	153 (113–200)	114 (81–161)	0.01**
*Type of anesthesia, n (%)*				
General anesthesia (GA)	25 (48.1)	17 (63.0)	86 (66.7)	0.073***
Conscious sedation (CS)	24 (46.2)	8 (29.6)	38 (29.5)	0.080***
Switch CS to GA	2 (3.8)	1 (3.7)	4 (3.1)	0.957***
Imaging characteristics				
CT/CTA *n*, (%)	51 (98.1)	27 (100)	98 (76.0)	**<** 0.0001***
CTP *n*, (%)	51 (98.1)	27 (100)	14 (10.9)	**<** 0.0001***
MRI *n*, (%)	1 (1.9)	1 (3.7)	33 (25.6)	**<** 0.0001***
*Infarct volume (ml)*				
*Median* *(IQR)*	26.7 (15–45)	56.4 (30–80)	NA (NA)	**<** 0.0001**
*Perfusion lesion volume (ml)*				
*Median* *(IQR)*	70.3 (40–132)	109.6 (59–146)	NA (NA)	0.169**
*Median ASPECTS at baseline, (IQR)*	8 (7–10)	8 (7–10)	8 (6–9)	0.148**
Site of symptomatic vessel occlusion^a^				
*MCA proximal*	39 (75.0)	20 (74.1)	95 (73.6)	0.973***
*Internal carotid artery (ICA)*				
Intracranial no T	5 (9.6)	5 (18.5)	11 (8.5)	0.290***
Intracranial T	11 (21.2)	5 (18.5)	36 (27.9)	0.454 ***
*Tandem occlusion*	5 (9.6)	3 (11.1)	2 (1.6)	0.015***
Risk factors				
Arterial hypertension, *n* (%)	37 (71.2)	21 (77.8)	100 (77.5)	0.509***
Blood pressure at admission (mm Hg)	151.7 (22.1)	161.6 (29.7)	152.4 (29.3)	0.301*
Diabetes mellitus, *n* (%)	10 (19.2)	4 (14.8)	28 (21.7)	0.491***
Hypercholesterolemia, *n* (%)	18 (34.6)	7 (25.9)	38 (29.5)	0.496***
Atrial fibrillation, *n* (%)	13 (25.0)	10 (37.0)	46 (35.7)	0.600***
Treatment				
rtPA treatment, *n* (%)	22 (42.3)	10 (37.0)	57 (44.2)	0.788***
Periinterventional anticoagulation	8 (15.4)	2 (7.4)	19 (14.7)	0.617***
Extracranial stenting, *n* (%)^c^	12 (23.1)	2 (7.4)	14 (10.9)	0.130***
Aspiration catheter only, *n* (%)	28 (53.8)	16 (59.3)	60 (46.5)	0.458***
Aspiration + stent retriever, *n* (%)	38 (73.1)	17 (63.0)	79 (61.2)	0.302***

*IQR* interquartile range, *NIHSS* National Institutes of Health Stroke Scale, *pmRS* premorbid modified Rankin Scale, *LSW* time patient last seen well, *CTP* computed tomography perfusion imaging, *rtPA* recombinant tissue plasminogen activator, *ASPECTS* Alberta Stroke Program Early CT scor, *MCA* middle cerebral artery

*p-values resulting from ANOVAs, **p-values resulting from Kruskal-Wallis analysis, ***p-values resulting from Pearson’s χ^2^-test. Values in bold indicate significant intergroup differences

****p-values after correction for multiple comparisons. Values in bold indicate significant differences at the 5% level of significance

*NA* no values available

^a^Multiple occlusion sites possible

^b^Note the overlap of 14 patients

^c^Stenosis ≥ 70%, no occlusion

### Imaging and Association with Clinical Outcome

Preinterventional imaging in DD and NDNDwP was based on CT including CTP only, except for one patient with additional MRI (including MR perfusion imaging). Within the NDNDnP cohort significantly more (*n* = 33, 26%; *p* = < 0.0001) patients were selected for late EVT based on MRI (without MR perfusion imaging). The median size of the estimated ischemic core volume in DD was significantly smaller when compared to NDNDwP (26.7 ml [15–45] vs. 56 ml [30–80]; *p* = < 0.0001), without significant difference in hypoperfused volumes between both groups.

**Fig. 2:**
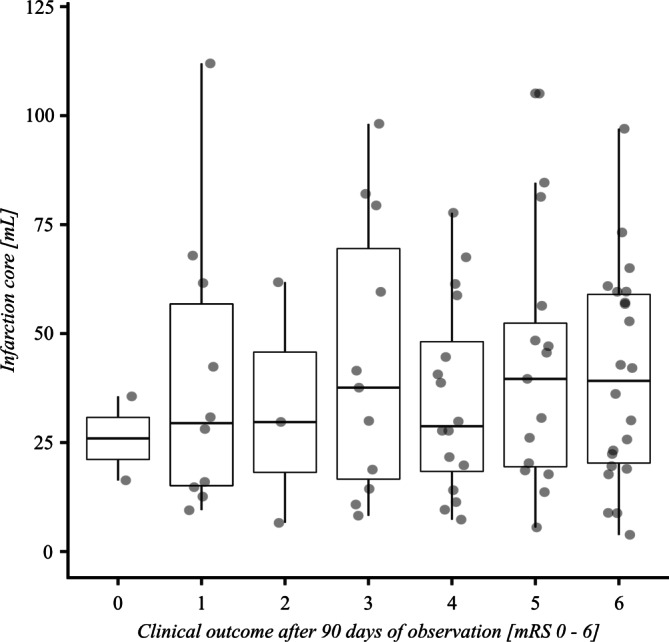
Estimated infarct volume (ml) in correlation to the results on the modified Rankin scale (mRS) at 90 days. The figure suggests a trend for positive linear association between the volume of the infarction core and mRS90; however this finding was not statistically significant (*p* = 0.673)

Fig. 2 suggests a trend for a positive linear association between the volume of the infarct core and mRS at 90 days; however, this finding was not statistically significant (Pearson correlation coefficient of 0.048; *p* = 0.673). No significant predictive value for good outcome (mRS 0–2) in the univariate analysis using 10 ml increment core volume could be found. Mismatch ratio > 1.8 presented an association with good clinical outcome (Odds Ratio [OR] 4.67; 95% confidence interval [CI] 0.57–38.34) whereas ischemic core > 70 ml (OR 0.44; 95% CI 0.05–3.74) as well as penumbra ≥ 15 ml (OR 0.51; 95% CI 0.13–1.92) were associated with an unfavorable outcome. The ASPECTS over all groups was without predictive association in univariate analysis. Within the DD cohort the ischemic core volume on CTP was significantly higher (*p* < 0.0001) in patients with an initial ASPECTS of 0–7 (median [25%, 75% quartile]: 73.2 [57.9; 97.6] ml) compared to patients with ASPECTS ≥ 8 (median, [25%, 75% quartile]: 39.15 [25.2; 61.7] ml).

### Clinical Outcome and Safety

**Fig. 3:**
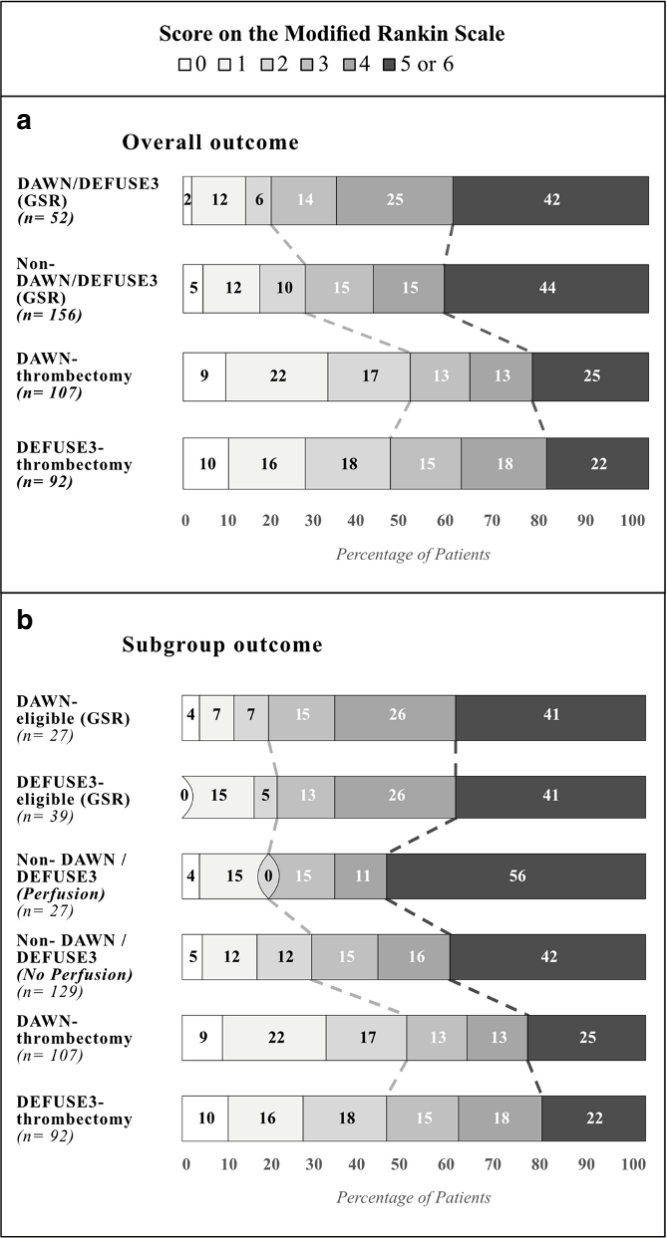
Distribution of scores on the modified Rankin Scale (mRS) at 90 days. Shown is the shift analysis of scores on the modified Ranking Scale within the GSR and its subgroups compared to the RCT’s original results. DAWN/DEFUSE3 (GSR) represents all trial-eligible patients that received ET plus standard medical therapy between 6 to 24 h. Non-DAWN-Non-DEFUSE3 (GSR) represents all trial-ineligible patients that received ET plus standard medical therapy between 6 to 24 h. DAWN-/DEFUSE3-thrombectomy are the RCT’s original results of the thrombectomy group. The numbers in the bars are percentages of patients who had each score; the percentages may not sum to 100 because of rounding. Scores on the modified Rankin scale range from 0 to 6, with 0 indicating no symptoms, 1 no clinically significant disability, 2 slight disability, 3 moderate disability, 4 moderately severe disability, 5 severe disability, and 6 death

**Fig. 4:**
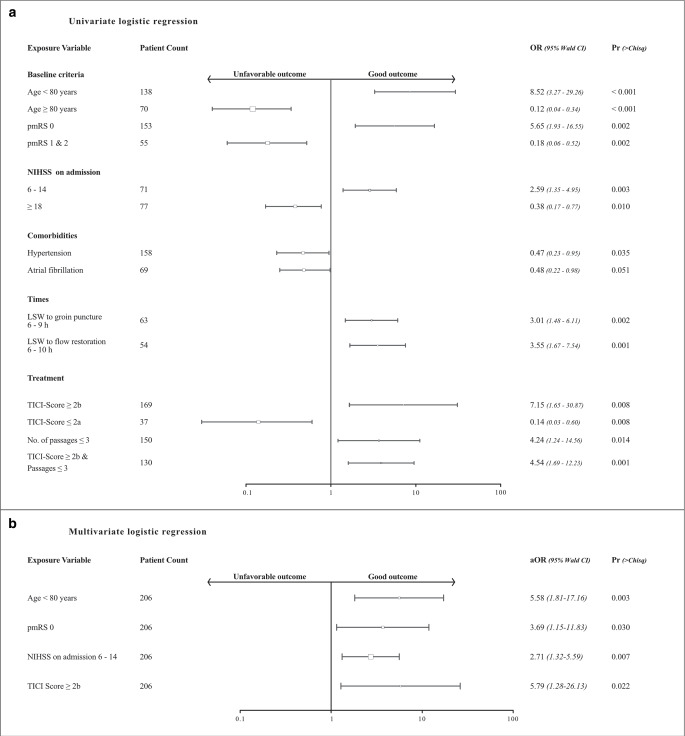
Subgroup analyses good clinical outcome. The forest plot in **a** shows the significant common odds ratio (OR) for good outcome (defined as a score on the modified Ranking Scale of 0 to 2 or back to baseline) at 90 days. Unfavorable outcome is defined as modified Rankin Scale 3–6. **b** shows the odds ratio adjusted for age, premorbid modified Ranking Scale 0 (pmRS 0), National Institutes of Health Stroke Scale (NIHSS), and modified thrombolysis in cerebral infarction (mTICI score). *aOR*, adjusted odds ratio. The size of the squares is proportional to the number of patients in the subgroup. *p* ≤ 0.05 was considered significant, *Wald CI* Wald confidence interval, *Chisq* Chi-squared test

The clinical outcome analysis, procedural results, and safety outcome is shown in Table 2 and Fig. 3. Overall mRS after 90 days did not present significant differences across groups. The best association for good clinical outcome in univariate and multivariate regression models showed age < 80 years (OR 8.52; 95% CI, 3.27–29.26), pmRS 0 (OR 5.65; 95% CI, 1.93–16.55), stroke severity on admission expressed as NIHSS 6–14 (OR 2.59; 95% CI, 1.3–4.95) and final mTICI score ≥ 2b (OR 7.15; 95% CI, 1.65–30.87). In univariate regression, an association between good clinical outcome and last seen well to groin puncture within 9 h (OR 2.38; 95% CI, 1.13–5.05) and time to flow restoration within 10 h (OR 2.15; 95% CI, 1.08–4.27) was present (Fig. 4).

**Table 2:** Outcomes at 90 days, procedural results, and safety outcome^a^

Characteristics	DAWN DEFUSE eligible (*n* = 52)	NON-DAWN NON-DEFUSE (with perfusion) (*n* = 27)	NON-DAWN NON-DEFUSE (no perfusion) (*n* = 129)	Overall *p*-value
Modified Rankin Score, median, (IQR)	4 (3–6)	5 (3–6)	4 (2–6)	0.807**
Good functional outcome (mRS 0–2), *n* (%)	10 (19.2)	5 (18.5)	36 (27.9)	0.343***
Mortality (mRS 6), *n* (%)	14 (26.9)	8 (29.6)	43 (33.3)	0.690***
ICH 24 h *n* (%)	6 (11.5)	9 (33.3)	21 (16.3)	0.049***
*Procedural results*				
mTICI 2b/3, % (*n*)	44 (84.6)	24 (88.9)	101 (78.3)	0.451***
First-pass rate, % (*n*)	22 (42.3)	8 (29.6)	47 (36.4)	0.699***
Number of passages, median, (IQR)	2 (1, 3.75)	2 (1, 3.25)	2 (1.3)	0.743**
Periprocedural complications, *n* (%)	9 (17.3)	5 (18.5)	16 (12.4)	0.622***

*IQR* interquartile range, *mTICI* modified thrombolysis in cerebral ischemia, *mRS* modified Rankin scale, *ICH* intracerebral hemorrhage

*p-values resulting from ANOVAs, **p-values resulting from Kruskal-Wallis analysis, ***p-values resulting from Pearson’s chi-squared test. Values in bold indicate significant intergroup differences

****p-values after correction for multiple comparisons. Values in bold indicate significant differences at the 5% level of significance

^a^Note the overlap of 14

Mortality was similar among the groups (*p* = 0.7). Intracranial hemorrhage (ICH) within 24 h was highest within the NDNDwP cohort (33%; *p* = 0.049), 16% in NDNDnP and lowest within DD (12%). A significant association of ICH with unfavorable outcome (OR 0.15; 95% CI, 0.03–0.64) was found in univariate analysis (Table 2).

## Discussion

### Prevalence and Outcome

Relative to all patients enrolled in the German Stroke Registry, the number of patients presenting in a late time window was 11%. Consistent with other studies [13–16], out of this cohort, only 25% of patients were DAWN/DEFUSE3 applicable. Thus, after the amendment of the strict advanced perfusion imaging inclusion criteria, the vast majority (75%) of late presenting patients would have been excluded from EVT because of missing perfusion imaging. These observations are supported by a recent study in which patients screened with routine CTP had a 41% reduced odds of undergoing EVT compared to a cohort identified based on NCCT + CTA and CTP only optionally, while no differences in clinical outcome were present [16].

For DAWN/DEFUSE3 eligible patients our results indicate inferior outcome for late thrombectomy compared to the RCT results. This applies to all patients in our cohort treated ≥ 6–24 h, and even for those patients who would have formally met the study criteria (mRS = 0–2: 20% in our cohort vs. 48% in DAWN and 44% in DEFUSE3). This is in line with a recent single-center analysis by Salam et al. (*n* = 166) where good functional outcome occurred in 24% [17]. The BEST cohort [9], another real-life study, also found worse clinical results of trial-eligible patients when compared to the randomized studies, but these were more favorable than in our cohort (mRS 0–2 = 30%, 14/47); however, the estimated infarct core volumes in BEST were very small (6 ml [0–20]) [9]. A very high proportion of patients with a good clinical outcome (67%) was found in a Swiss study including 52 ET patients [7] but the majority of patients (71.1%) in this study had M2 occlusions, which are in general more often associated with a good clinical outcome [18]. A series with MRI-based patient selection (no perfusion parameters) yielded results comparable to those of the randomized studies [19]; however, all of these real-life studies have not been able to differentiate precisely between proximal and distal MCA occlusion types, which significantly affects the prognosis, whereas only angiographically proven ICA and proximal MCA occlusions were included in our series. This certainly contributes at least in part to the significantly higher estimated infarct cores before treatment and poorer outcome results of our study when compared to other real-life study results.

Furthermore, the aim of DAWN and DEFUSE3 was to show that thrombectomy as a treatment modality can be effective in the late time window of stroke. Inclusion and exclusion criteria of these RCTs were chosen accordingly. From a clinical perspective, however, our aim as clinical physicians is to offer each patient the best medical care. In some cases, this may mean that we treat patients with low chance of good outcome (e.g. low ASPECTS), although these patients would not have been included in DAWN/DEFUSE. This may lead to different results of RCTs vs. real-world data.

For our DAWN/DEFUSE3 ineligible patients favorable clinical outcome at 3 months was slightly lower (26%) when compared to a recent secondary analysis of DEFUSE3 patients (32%; 48/149), who had an overall small core volume (7.3 ml [0–14]) but did not meet DAWN criteria [4]. In line with our results, in a subgroup of those patients with a significantly larger core volume (45.2 ml [37–60]) and thus ineligibility for both trials, the percentage of good outcomes dropped to 24% (8/33) [4]. Within the BEST registry, 36% (16/45) of trial ineligible patients reached a good outcome, but NIHSS on admission (12 [7–18]) and core volume (15 ml [0–49]) were much lower compared to our cohort, possibly explained by the high rate (41%) of M2 occlusions.

### Patient Selection: Clinical and Imaging Parameters

In our evaluation there was a tendency towards a negative correlation between the extent of estimated infarct core and the final clinical outcome but in contrast to other studies [20], we were not able to define a clear limit of the infarct volume, above which a good clinical outcome becomes unlikely. Based on imaging only, we are currently unable to accurately determine whether tissue is already irreversibly damaged or not. Thus, a critical re-evaluation of the core concept as it is used in clinical practice has been proposed [21] as neither the absolute volume of the infarct core nor the collateral status are the only factors that determine the clinical outcome of revascularization treatment [22, 23]. On the other hand, the benefit of EVT for patients with little penumbra and an already large existing infarct core is uncertain [24]. Overall, our patients had significantly larger infarct cores when compared to other studies, which in turn might have led to an increased number of hemorrhagic complications and an overall poor outcome (mRS 5 and 6) of more than 50% in patients with estimated pretreatment core infarcts above the upper limits of the trial. Ongoing trials assessing outcomes after EVT in patients with pre-existing large cores and optimal imaging selection criteria will address these open questions (https://www.clinicaltrials.gov; Unique identifier: NCT03094715, NCT03805308, NCT03876457).

In the analysis of MR CLEAN and the HERMES pooled registry, in which most patients were selected based on NCCT, often in conjunction with an ASPECT score and CTA, lower ASPECTS had worse outcome, but still had a benefit from thrombectomy [25]. In our cohort, the ASPECT score alone was not a predictive factor for good clinical outcome.

In a subgroup analysis of DEFUSE3, endovascular recanalization was significantly more often associated with an improved outcome when the patient selection was done by MRI (OR 11.9; 95% CI, 2.2–63.4) compared to CTP (OR 6.1; 95% CI, 2.2–17.1) [5]. In our study, 26% (33/129) of patients who did not meet the DAWN/DEFUSE3 criteria and did not undergo CTP (NDNDnP group) were selected by MRI (DWI-FLAIR mismatch). This may contribute to the higher number of good clinical results in this specific group and could be an alternative to perfusion imaging, even though there is evidence that extended window patients may be safely treated even in the absence of CTP or MRI data and CTP acquisition is not associated with better outcomes [16, 26].

### Clinical Parameters

In accordance with previous studies, baseline stroke severity on the NIHSS was a powerful independent predictor of clinical outcome [2, 5, 25]. Almost all patients in DAWN (98%) and most patients in DEFUSE3 (87%) had no pre-existing neurological deficits compared to our study (77% DD and 63% in the NDNDwP). But the premorbid mRS status was strongly associated with good clinical outcome and is therefore another important clinical factor which should be considered in the decision for or against endovascular treatment [27]. Compared to the randomized trials the overall percentage of patients > 80 years in our cohort was higher (33% in DD and NDNDnP and 41% in NDNDwP vs. 23% and 24% in DAWN and DEFUSE3), pointing out another important factor for patient triage in clinical practice. The benefit of EVT has however shown to be similar between age groups [5]. Following these results, age < 80 years was a strong predictor for a good functional outcome at 3 months [5, 25]. Numerous studies have shown that age and pmRS play a crucial role in the outcome after stroke and could be another important explaining factor for the inferior outcome of our cohort compared to the RCT results.

### Treatment Factors

In unadjusted analysis, the type of anesthesia was not associated with higher rates of good clinical outcome. Results of previous reports are controversial regarding general anesthesia (GA) versus conscious sedation (CS) [28]. Considering that most patients in our cohort were treated under general anesthesia, factors such as arterial hypotension, effects of the anesthetics, and others may have contributed to the comparatively poor clinical outcome of our study in addition to the factors discussed [29].

### Times/Number of Passages

Even though the treatment effect persisted over time in DEFUSE3 [5], the risk ratio for unfavorable outcome increased in patients with a time window of > 9 h between onset and randomization [3] and in HERMES the treatment effect weakened over time and was no longer statistically significant > 7 h [30]. This is consistent with our findings of a significant association of shorter time to recanalization with favorable outcome. It is worth noting that the best outcomes in our study were in the NDNDnP group which was up to 40 min faster from arrival to reperfusion. Patients requiring ≤ 3 thrombectomy passes had more substantial reperfusion and favorable outcome at 3 months [25].

### Limitations

The main strengths of our study are the multicenter design and the strict application of DAWN and/or DEFUSE3 study criteria; however, the results are based on observational data, which are subject to well-known limitations (e.g. no untreated control group). The strict application of study criteria (e.g. artery occlusion types) resulted in a limited dataset. Furthermore, of the 729 excluded patients due to ≥ 1 relevant missing datapoint, 92 were in the late time window (> 6 h) potentially eligible for DAWN or DEFUSE. Given the multi-investigator design perfusion imaging protocol was not standardized and technical and mathematical differences in software packages cannot be fully overcome [31], which might influence the estimation of ischemic core and hypoperfusion compared to RAPID software; however, all measurements were centrally re-evaluated at a single institution using the same software for all data simulating the threshold parameters used by the RAPID (iSchemaView Menlo Park, CA, USA) software.

## Conclusion

Our data show that in clinical practice more liberal selection criteria are used. The high rates of good functional outcome known from the randomized trials on late thrombectomy are not achieved, irrespective whether the strict inclusion criteria are applied or not. The strict application of the DAWN/DEFUSE3 inclusion criteria would exclude a high number of patients from treatment even though patients triaged without perfusion imaging had the shortest arrival to reperfusion times, and the highest rates of independence at 90 days. Alternative selection criteria in late thrombectomy might increase the likelihood for EVT and should be studied further.

## Supplementary Information

Supplementary Information contains morge detailed information on methods used for image acquisition and analysis aswell as static methods used.
