# Supplementary material for: Late Thrombectomy in Clinical Practice: Retrospective Application of DAWN/DEFUSE3 Criteria within the German Stroke Registry
Source: Clin Neuroradiol. 2021 Jun 7;31(3):799–810. doi: 10.1007/s00062-021-01033-1 (PMC8463374; doi:10.1007/s00062-021-01033-1)
Supplement: Supplementary file 1 — Supplementary Information contains morge detailed information on methods used for image acquisition and analysis aswell as static methods used. [file 62_2021_1033_MOESM1_ESM.pdf]

Online Data Supplement:

Methods:

Image Acquisition and Analysis

Local imaging protocols varied but included at least a non-contrast-enhanced CT (NCCT) scan and a CT angiogram (CTA) for baseline imaging. CT Perfusion Imaging (CTP) and Magnetic Resonance Imaging (MRI) with and without perfusion could be used additionally. Evaluation of ASPECTS and mTICI score was performed by the local neuroradiologists. Infarct volumes were centrally measured for all patients by KS and MH on CTP or on MRI if available. In order to overcome technical problems due to different scanners, imaging protocols and software, perfusion maps of cerebral blood flow (CBF) and cerebral blood volume (CBV) were created from the original raw data using the syngo.via Volume Perfusion Neuro commercial package (Siemens Healthineers, Germany). The volumetric infarction analysis of the ischemic core volume (ICV) and ischemic penumbra volume was achieved by using the approach of RAPID Software (iSchemaView)[1–3] as follows. Head motion artefacts and, if necessary, revision of timing with respect to the arterial input function (aIF) was performed. The threshold for estimating the size of the infarct core was assessed by using the regional cerebral blood flow (rCBF) on a deconvoluted CBF map. The infarct core was calculated by comparing the rCBF of the corresponding contralateral area to the affected area. A value below 30% of the physiological blood flow defined the infarction core. The penumbra volume was established with a Tmax deconvoluted map and an increase of density from a time of 6 seconds and higher (Tmax >6s).

Statistics:

Data were initially assessed for normality with the Shapiro-Wilk-Test and by visual inspection of their histograms. Based on these results, we used one-way analysis of variance (ANOVA) for the variables age and blood pressure at admission to test for equality of mean values in each data group. Inter-group differences in the remaining discrete and continuous, but not normally distributed variables (e.g. ASPECTS score, infarct volume size), were tested with the Kruskal-Wallis test. When statistically significant differences occurred, single post-test comparisons were performed by either using the t-test or the Mann-Whitney U test with Bonferroni correction for multiple comparisons, respectively. For binary or categorial variables (e.g. preexisting diabetes mellitus), contingency tables and $\chi2$-tests were used to detect differences between the groups. Post-hoc 2x2 tables were created and p-values were adjusted with Bonferroni correction.

Univariate binary logistic regression analysis was used to determine if a single parameter was able to differentiate between patients with poor and good functional outcome (mRS 0-2) at day 90. The independent variables tested included those that have previously been shown to affect outcome (age, NIHSS score, sex, systolic blood pressure, lesion volume, TICI score, number of passages) (Figure S2, supplemental Appendix). Odds ratios as estimates of relative risk with 95% confidence intervals (CIs) were obtained for each independent predictor. Wald statistics was used to determine whether a single predictor variable was statistically significant or not at $\alpha < .05$. Multivariate binary logistic regression analysis was performed to determine the independent parameters that could best predict the functional outcome. All parameters with a p-value less than 0.05 in the univariate binary logistic regression analysis were included for multivariable logistic regression analysis. However, not all those significant variables could be included to maintain statistical power. By use of the variables age > 80, pmRS = 0, NIHSS on admission 6 - 14 and a final TICI score of ≥ 2b a total number of 206 observations could be

maintained. A collinearity diagnosis was made on the multivariate logistic regression model to test the independence of predictors.

Differences in the distribution of the mRS90 scores in the subgroups of our study and the DAWN/DEFUSE3-thrombectomy groups were assessed with contingency tables and $\chi 2$-tests. Post-hoc Fisher's exact approach was used to compute the exact p-value for each cell[4]. The Pearson correlation coefficient (r) was used to interrogate the significance and strength of correlation between infarction core and mRS90. Mann-Whitney U-tests were used to compare the ischemic core volume between patients subdivided in groups with unfavourable and better ASPECTS.

Analysis were performed using R (version 3.6.1 / R Core Team (2019) / https://www.R-project.org/) on R-Studio Version 1.2.5001 and the SPSS statistical package (IBM Corp. Released 2017. IBM SPSS Statistics for Windows, Version 25.0. Armonk, NY: IBM Corp.). A level of significance of alpha=0.05 was used throughout the study.

References

1. Cereda CW, Christensen S, Campbell BCV, Mishra NK, Mlynash M, Levi C, et al. A benchmarking tool to evaluate computer tomography perfusion infarct core predictions against a DWI standard. J Cereb Blood Flow Metab. 2016;36:1780–9. doi:10.1177/0271678X15610586.
2. Olivot J-M, Mlynash M, Thijs VN, Kemp S, Lansberg MG, Wechsler L, et al. Optimal Tmax threshold for predicting penumbral tissue in acute stroke. Stroke. 2009;40:469–75. doi:10.1161/STROKEAHA.108.526954.
3. Albers GW. Use of Imaging to Select Patients for Late Window Endovascular Therapy. Stroke. 2018;49:2256–60. doi:10.1161/STROKEAHA.118.021011.
4. Shan G, Gerstenberger S. Fisher's exact approach for post hoc analysis of a chi-squared test. PLoS ONE. 2017;12:e0188709. doi:10.1371/journal.pone.0188709.
